# Self-dehumanisation in voice hearers: the end of a continuum

**DOI:** 10.3389/fpsyt.2023.1173380

**Published:** 2023-10-03

**Authors:** Bethany O’Brien-Venus, Tom Jenkins, Paul Chadwick

**Affiliations:** Department of Psychology, University of Bath, Bath, United Kingdom

**Keywords:** voice-hearing, thematic analysis, self-dehumanisation, meta-dehumanisation, psychotic phenomena

## Abstract

**Background:**

Meta-dehumanisation and self-dehumanisation have been identified as potentially relevant phenomena for developing a deeper understanding of distress related to voice-hearing, particularly those experiencing voices as part of psychosis. Chadwick has previously argued that those with psychosis, including those who hear distressing voices, typically feel “dehumanised and set apart by their experiences of psychosis and trauma.” The present study explores the subjective experience of self-dehumanisation in people who experience distressing voices, which was selected as a useful starting point to inform future research focused on understanding dehumanisation in people with psychosis.

**Methods:**

Qualitative data was obtained through twenty semi-structured interviews with self-identifying voice hearers and analysed using reflexive thematic analysis. This followed the recursive six phase procedure of Braun and Clarke, and this was conducted from a critical realist, contextualist position.

**Results:**

Reflexive thematic analysis of participant’s experiences produced a core theme, Dehumanisation as the End of Experiential Continua, and six subthemes: Extent of Distressing Sensory Fragmentation; Sense of Belonging with Other Humans; Integrity of Self as a Private, Coherent Entity; Sense of Worth as a Human Being; Strength of Personal Agency; and Trust in Own Credibility and Reliability. Two further themes, The Push and Pull of Dehumanising Forces and Reclaiming Life through Humanising Forces, were identified. Findings were presented to a panel of five experts by experience, all with lived experience of psychosis and service-use; all five strongly endorsed the themes as fitting with and expressing their own experiences of self-dehumanisation.

**Conclusion:**

Reflexive thematic analysis of voice hearers’ accounts identified self-dehumanisation as the endpoint where six experiential continua coalesce. Experiential movement along these continua was affected by a range of interpersonal, intrapersonal, and societal forces over time, including dehumanising attitudes of others and voice malevolence and omnipotence. Future research might examine if and how psychological therapies aimed at those experiencing distressing voices, such as people experiencing psychosis may address feelings of self-dehumanisation.

## Introduction

“I’ve always thought that only nasty, horrible people would have voices, that’s why I’ve got voices, because you are just not a human being, you are not worthy of not having voices” [Sue, a voice hearer, quoted in (1)].

Dehumanisation is theoretically defined as the attitude or perception of another person or group as less than human (2) and excluded from the moral consideration the rest of humanity warrants (3). Being seen as less than human can be with respect to uniquely human (animalistic dehumanisation) or essentially human (mechanistic dehumanisation) characteristics, the former including self-control and rationality and the latter including emotionality, warmth, and agency (2). A recent alternative theory is that dehumanisation is the cognitively dissonant attitude that another person is simultaneously less than human but also still human in some respects, albeit uncannily or dangerously so (4).

Meta-dehumanisation is the perception of being dehumanised by others, for instance, believing that others perceive you, or a group you belong to, as lacking essentially or uniquely human characteristics (5, 6), and self-dehumanisation is the self-perception of being less than human (7). These concepts from social psychology have been usefully applied in mental health research, for instance in examining the relationship between stigma and dehumanisation in individuals with alcohol-use disorders. Stigma is defined as the experience people have when “individuals possess (or are believed to possess) some attribute, or characteristic, that conveys a social identity that is devalued in a particular social context” (8). For example, research with alcohol-use disorders found that stigma awareness is associated with meta-dehumanisation, and self-stigmatisation is closely aligned with self-dehumanisation (9); and that self-dehumanisation mediates the relationship between meta-dehumanisation and increased anxiety, depression, and decreased drinking refusal self-efficacy (10). This research was the first of its kind to apply the concept of self-dehumanisation to mental health research.

Likewise, there are reasons to believe that meta-dehumanisation and self-dehumanisation may be relevant phenomena for developing a deeper understanding of distress related to the phenomenon of voice-hearing in psychosis and schizophrenia-spectrum disorders. Of all people diagnosed with schizophrenia-spectrum disorders, 60–80% experience auditory hallucinations (11). In Western society, psychosis and schizophrenia-spectrum disorders are associated with the most negative stereotypes and lowest expectancy of recovery (12) and stigmatising representations continue to be prevalent in the media (13). An American study found that people with a diagnosis of schizophrenia were perceived as significantly less human than those from a non-clinical population, as well as those with other mental health conditions, such as depression and anxiety (14). Chadwick (1) argues that people who hear distressing voices typically feel ‘dehumanised and set apart by their experiences of psychosis and trauma’. Furthermore, individuals with early psychosis tend to perceive themselves as inferior to and of lower social rank than matched controls (15), suggesting the possibility of perceiving themselves as less human than others. It is also possible that everyday experiences of dehumanisation could directly contribute to the onset and maintenance of post-psychotic depression and social anxiety, given the relationship of these to experiences of entrapment by voices, humiliation, shame, and social marginalisation (16, 17). Overall, the research in this area suggests that the experience of dehumanisation may be a relevant phenomenon in these populations.

Thus, specifically exploring the experience of dehumanisation in people who hear distressing voices may be a useful springboard for future research exploring the experience of dehumanisation in people with psychosis and schizophrenia-spectrum disorders, as it is possible that the relationship such individuals have with their voices is dehumanising (18), in addition to any societal or interpersonal dehumanising processes. Also, the distress psychotic voice-hearers’ experience has been linked to their appraisal of the degree of omnipotent power voices’ hold and the consequent sense of entrapment by their voices (19). This is supported by the finding that degree of subordination in relation to a voice often parallels subordination in social relationships (20), and such subordination may relate to experiences of dehumanisation.

### Aim

The present study was aimed at understanding what constitutes the experience of feeling dehumanised in people who hear distressing voices and what factors influence the development and mitigation of this experience, with a view to opening the pathway to future research which may explore the role of dehumanisation in people who hear voices within the context of psychosis. It aimed to provide a foundation of understanding the phenomenon of dehumanisation in a broad sample of participants experiencing distressing voices.

### Research question

How do voice-hearers describe the subjective experience of feeling dehumanised and, conversely, the experience of feeling humanised?

## Methods

### Design

This research uses reflexive thematic analysis of qualitative data gathered through individual semi-structured interviews. The interview schedule was developed and revised through discussion between the authors. It was piloted with and reviewed by a person with lived experience of voice-hearing as part of psychosis. The semi-structured interview questions were modified in accordance with the feedback given by the person with lived experience.

The research has been quality assessed against Braun and Clarke’s tool for evaluating reflexive thematic analysis (21). The ontological and epistemological stances were decided at the proposal stage and the analysis was conducted in a theoretically coherent manner with the selected stances. A critical realist, contextualist stance was adopted. The critical realist ontological stance proposes that a real world exists, however, it is only knowable through subjective, situated perception (22). Compatibly, the contextualist epistemological stance proposes that all knowledge arises from a context, which includes the era and society in which the research was conducted, and the researchers’ own subjectivity. It suggests that knowledge can be provisionally true within a given context (23). The analysis assumed the lived experience of participants was real but that access to this was only possible through the inherently subjective lens of the researcher. It also assumed that, within the context the specific research was conducted, it could produce provisionally true results.

### Participants

Participants were recruited using convenience sampling from Hearing Voices Network groups, the research advertising platform MQ Participate, and Twitter. Convenience sampling outside of NHS settings and minimally restrictive inclusion criteria were used to recruit a heterogeneous sample.

The inclusion criterion for this study was: currently self-identifying as experiencing at least one distressing voice. The exclusion criteria for this study were: being below the age of 18 at the time of recruitment and being unable to speak English. The criterion related to language was in place due to a shared understanding of language and meaning being important for reflexive thematic analysis. No criterion was in place regarding diagnosis and data were not gathered regarding whether any diagnosis had been received. The target analysis sample was 15–20 participants. A larger sample size was selected as the information power of the sample was expected to be lower due to low sample specificity, a cross-case analysis method, and broad research questions (24), while the use of semi-structured interviews and a blend of inductive and deductive coding using established theories of dehumanisation and voice-hearing was expected to enhance information power.

All participants were given an electronic copy of the information sheet and had the opportunity to ask questions prior to participating. Informed written consent was obtained for all participants via an online system, Qualtrics.^1^ Participants completed the BAVQr (25, 26) at the time of interview to screen for and confirm voice-related distress. Following interview completion, all participants received an electronic debrief sheet and a £10 voucher to compensate for their time.

### Data collection

Interviews were conducted remotely and audio-recorded, eighteen via video call and two via telephone, based on participants’ preference. Interviews were conducted and transcribed by the primary researcher (BO’B-V). The wording of the interview schedule, information sheet, and consent form were reviewed with a Person with Personal Experience of voice-hearing as part of psychosis to improve accessibility and sensitivity and minimise any potential distress due to the emotive and sensitive nature of the research topic. Data collection took place May–September 2021.

### Ethical statement

This study was given ethical approval by the University of Bath Psychology Research Ethics Committee (PREC Reference: 20-249; February 2021). The authors have abided by the Ethical Principles of Psychologists and Code of Conduct as set out by the British Association for Behavioural and Cognitive Psychotherapies (BABCP) and the British Psychological Society (BPS). All participants were provided with the information sheet, given an opportunity to ask questions, gave written consent, and consented verbally at the start of their interview.

### Reflexive thematic analysis

Braun and Clarke’s (27) six-step recursive and reflexive procedure was followed for the analysis. Reflexive thematic analysis was selected as it permits a theoretically informed research question with an experiential focus alongside a blend of inductive and deductive coding (27, 28). This was suitable given the pre-existing rich literature investigating voice-hearing in psychosis, as well as the growing literature theorising and exploring dehumanisation. A further strength of the method was its ability to explore recurrent patterns of meaning across a heterogeneous sample, while noticing instances of individuality.

The analysis was conducted using NVivo Version 12 (2020). The themes developed were analytical rather than descriptive and each theme and subtheme was comprised of both inductive and deductive codes.

The primary researcher familiarised with the transcripts through multiple readings, annotating, and two iterations of coding. Initial themes and subthemes were developed and mapped through discussions between BO’B-V and PC about the clustering of codes.

Following this, BO’B-V reviewed the clustering of codes under themes for a second time, merged two subthemes, and revised the theme names. BO’B-V moved recursively backwards and forwards between the interviews, extracts, codes, subthemes, and themes to check the evidence for the themes and subthemes, their boundaries and coherence.

Theme definitions were written for each theme and subtheme to outline their central organising concept and boundaries. Finally, theme names were refined, extracts were selected and woven into the narrative of the results.

### Reflexivity

BO’B-V had some experience of working with voice-hearers clinically in inpatient and community settings and had previously completed historical research into the recovery movement for those with psychosis. TJ had some experience working with voice-hearers in an inpatient setting and conducts research on the experience of dehumanisation in psychosis. PC had extensive experience of working with psychotic voice-hearers clinically and through research. All researchers felt curiosity and concern about the subjective experience of dehumanisation in voice-hearers.

### Validity, generalisability, and transferability

Final theme checking was completed by BO’B-V to verify the validity of themes against the content of interviews. Throughout the analysis, BO’B-V kept a reflexive log which recorded positions, assumptions, and influences throughout the research process.

BO’B-V sought to enhance analytic generalisability by developing a theoretically oriented analysis (28), identifying analytic themes that are relevant to all or many participants through blended inductive and deductive coding (29). The context, participants, and circumstances of the study are described such that the reader is enabled to assess the transferability of the research to other contexts (28).

To check the analysis and assess external validation within a clinical psychosis population, themes and subthemes from the present study were presented to an Expert by Experience panel of five people with lived experience of distressing psychosis who had previously accessed mental health services. The group was asked to provide their perspective on whether the themes fully captured and described their experiences of dehumanisation.

## Results

### Participants

Twenty voice-hearers participated, eleven men and nine women (none identified as non-binary). Eight participants identified as Black British, four as White British, two as Mixed (White and Black) British, one as Mixed (Black British and Chinese), two as Asian Indian, two as Asian Other, and one as White Other, European. Eleven participants were 18–24, four were 25–29, one was 30–34, one was 40–44, one was 50–55, one was 60–65, and one was 70+. Nineteen participants disclosed how long they had heard voices for and completed the BAVQr. Participants reported having heard voices on average for 11 years, with a range of 1–70 years. At the time of study participation, median BAVQr scores were Malevolence (8), Benevolence (7), Omnipotence (9), Resistance (18), and Engagement (7). 47% of participants scored 10 or more on either Omnipotence or Malevolence, and 37% of participants scored 10 or more on *both* voice Malevolence and Omnipotence plus 16 or more on Resistance (these are consistent with scores seen in clinical samples of voice hearers with confirmed diagnoses of psychosis, e.g., (25, 26)).

The average length of an interview was 37 min (range 17–82 min). One participant was recruited via the Hearing Voices Network, four participants were recruited via Twitter, and fifteen via MQ Participate. A further fourteen potential participants expressed interest but did not respond to invitation to schedule an interview.

### Themes

**Figure 1:**
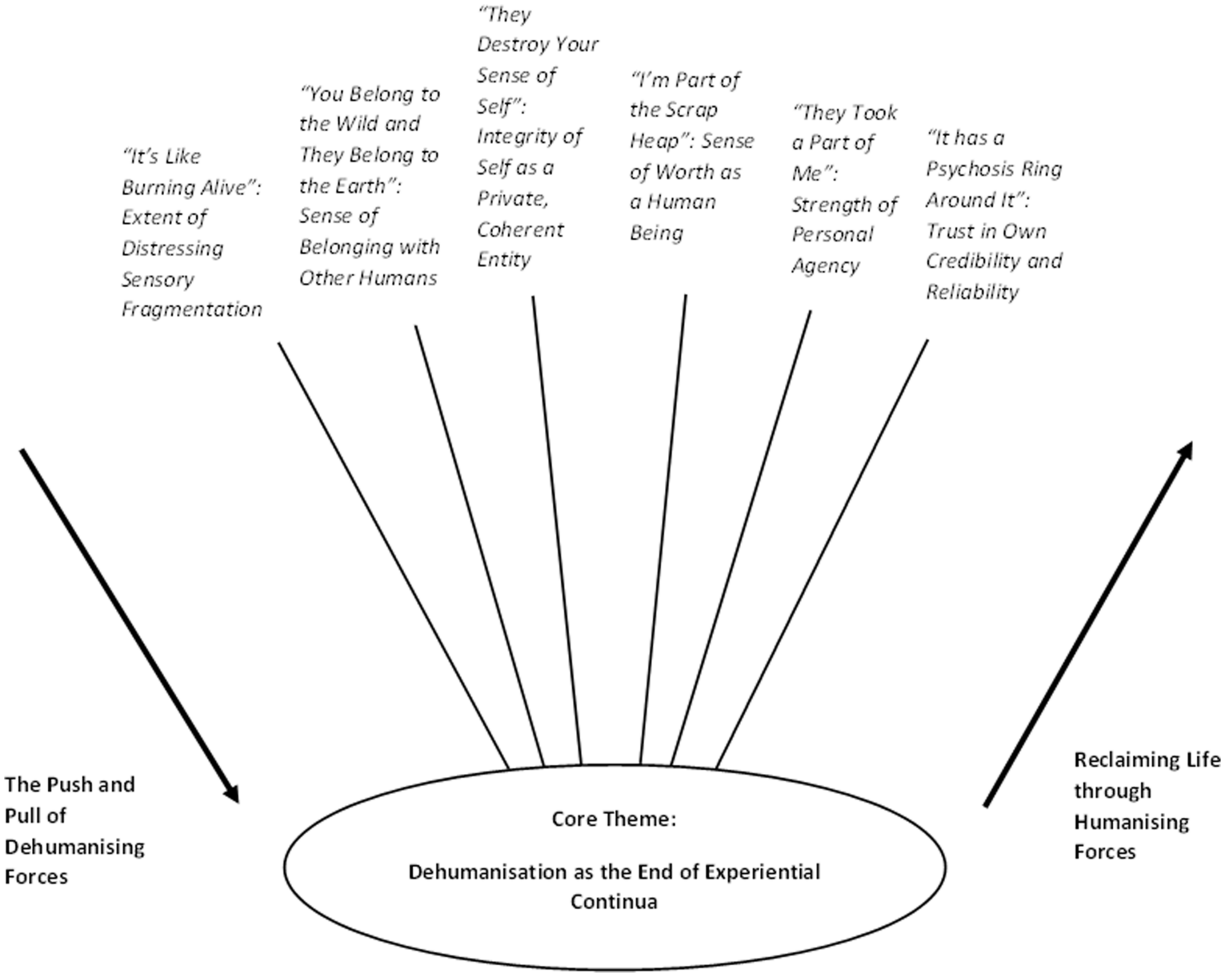
Map of themes and subthemes.

Six different kinds of experiential changes were identified as constituting feeling dehumanised for voice-hearers. These changes represented the loss of some essentially human quality or capability. Feeling humanised involved these changes reversing. A range of societal, interpersonal, and intrapersonal forces were shared by participants as either moving them towards feeling dehumanised or moving them away from this. See Figure 1 for the map of themes and subthemes and Supplementary Table S1 and Appendix I for the codes underpinning these.

#### Core theme: dehumanisation as the end of experiential continua

Voice-hearers’ descriptions of feeling dehumanised indicated six key psychological processes (sub-themes): that is, distressing sensory fragmentation, lower self-worth, lower sense of belonging with others, less sense of themselves as private and coherent, weaker personal agency, and loss of trust in themselves and their credibility. There was substantial intra-and inter-personal variation within each, and hence these six subthemes were conceptualised as being like continua.

A central unifying core theme emerged, that of Dehumanisation as the End of Experiential Continua. Thus, whilst for different participants, different combinations of the six sub-themes were more important than others, in moments of self-dehumanisation, participants experienced a facet of each continuum, like rivulets coalescing into one stream. And there was a strong and consistent sense that dehumanisation was the end of each continuum, the end of the line so to speak.

Two further themes were developed which related to humanising and dehumanising forces – that is, forces which moved people along continua either towards or away from feeling dehumanised.

##### “It’s like burning alive”: extent of distressing sensory fragmentation

Participants expressed that voice-hearing often felt inexplicable and terrifying at first and that this contributed to feeling, at worst, not human for having “lost it” (P12). The experience of voice hearing was often perceived as alien initially, and for some continued to feel alien to the self and difficult to reconcile, as P1 said, “these are alien voices that I’m hearing, they are not my own voice […] they do seem to know what’s going on in my mind, but they are still alien to me.”

Some participants reported extreme distress with strong sensory qualities, with one participant describing voice-hearing as like a “traumatising attack” (P17), and another describing the intensity as “like pouring out of my skin […] it’s like burning alive” (P8). Others reported that, during their recovery, they managed to start experiencing their voices as a harmless event in the mind rather than as having “some sort of god-like quality to them so they sort of felt kind of all powerful and difficult to resist,” which related to starting to feel human again:

I can now say to the men, you know, I don’t believe you or, you know, if they’re making a threat, I can say, “ah, that’s just an empty threat, you can’t follow through on that (P4)

##### “You belong to the wild and they belong to the earth”: sense of belonging with other humans

Voice-hearers reported feeling alone with the experience of voices, alongside feeling rejected from valued social groups or from society. The feeling of not being acceptable to other people contributed to a sense of defeat and thoughts about removing the self from humanity, or already being in some way removed:

When you try to fit into the society and to your friends and you try your level best to be the one person they used to know before the voices came […] and it doesn’t matter the efforts you make the people they’re just like “no, no, no, we cannot accept you here” […] it makes you feel like you’re not a person anymore, like you belong to the wild and they belong to the earth, and you are in the wild, you are just one, you are just you, you’re not a person, you’re no one else (P12)

One participant (P20) reflected that he perceived himself as belonging towards the bottom of the hierarchy of beings and felt he could never move up this hierarchy regardless of what he did: “I’ve just come to the understanding that I’m just less of a person because of it, I’m the subset of the subset of people.” He noted the importance of his intersection here of hearing voices and being from a minority ethnic group, which he felt compounded his position.

Others believed that their struggle to match the behaviour and achievements of others deprived them of belonging with other humans:

[I] view myself like less of a person because I can’t make decisions like a normal person, I can’t carry on with my life, I can’t do the same as other people, I feel like undignified, I feel like I’m not fit to be alive (P19)

##### “They destroy your sense of self”: integrity of self as a private, coherent entity

Voice-hearing impacted on participants sense of the integrity of their self and identity, with this initially feeling destroyed or diminished for some. Those with more malevolent or incongruent voices experienced them as more destructive towards their sense of self. This loss of a sense of a coherent, integrated self contributed to feelings of being less of a person or not quite human, as P4 reported, “the thing I always emphasise about these kinds of experiences is how initially they completely destroy your sense of self,” and as described by P14, “I cannot do what I feel like, like dancing, it has eliminated me from dancing which was my which was my hobby and my passion […] it has really diminished my personality […] really affected my character and my reputation.”

Some described feeling that their mind was no longer a private place and felt a strong sense that their voices could abuse access to their mind, or impact on activities which were integral to their sense of self, as indicated by P19, “they just heard my decisions, what I want to do, and they do contrary.”

##### “I’m part of the scrap heap”: sense of worth as a human being

Many participants described feeling a loss of self-worth due to their experience of voice-hearing and associated impacts, as P15 expressed, “most of the time the voices dehumanise me I feel like I’m not enough.” P20 highlighted a similar feeling:

I’m not gonna leave the scrap heap because I know I can’t really, if I think about it my reality is the scrap heap, but I will be at the top of the scrap heap rather than towards the bottom […] for me it was very much I’m already not human, as it is, I’m just this thing (P20)

Some reported feeling exhausted and defeated by the constant fight to prove their own worth, with the eldest person in the study (P1) reporting that this fight had continued for decades by saying, “I always have to challenge myself to believe that I am really a worthwhile person. […] I have to sort of deploy arguments like that to prove that I’m not a worthless person, as the voices keep insisting.”

Others noted feeling inadequate as a human being compared to other people and feeling less capable than others, and some perceived their voices and other people as in agreement about the kind of criticism they deserved:

The men would be swearing at me telling me that I was rubbish in various flowery language sometimes commanding me to kill myself so […] yeah almost kind of taking on board and believing what they were saying about me you know internalising all of that (P4)

##### “They took a part of me”: strength of personal agency

Voice-hearers shared feeling a loss of control or influence over their lives and an impact of hearing voices on how strong they perceived their personal agency to be:

I didn’t want to do this, they took a part of me […] why do you have to act like that lunatic if you say you are not? When you’re trying to defend yourself, you yourself just say yourself like “okay I think I’m a lunatic now, because I haven’t controlled it” (P10)

Experiencing voices taking over their actions and choices or reducing their ability to perform valued behaviours and activities contributed to this reduced sense of agency. P1 reported needing to exert high levels of focus to stop himself performing behaviours his voices wanted him to do, for example, “I concentrate hard as I’m able to do and force myself not to do it […] I lose a bit on the swimming front, because I never swim out of my depth.”

Experiencing fighting the voices as ineffective further weakened people’s sense of their own agency. Many noted impacts on their functioning in valued areas of their lives and some felt unable to meet their own expectations as well as those of the voices, as P9 said, “You have tried everything every possibility not that you cannot, you cannot achieve it you feel like demoralised you feel low […] you feel less of a human.”

##### “It has a psychosis ring around it”: level of trust in own credibility and reliability

Voice-hearers cited losing trust in their own credibility, believability, and decision-making capabilities, through the influence of voices, the control voices have over their actions, and through the “psychosis ring” (P20) other people place around what they say and do. This contributed to a feeling of not being human, for instance, for P8, “the guilt, the frustration, the assessing my life, and they just um I just perceive myself as the worst, worst creature in the planet really.”

One participant described failing to catch a rat leading to this kind of doubt in their mind:

Because there wasn’t anything […] nothing came in the front room, it’s like my brother says to me, “Well, you know, it’s because of your illness” and I’m kind of going, “Is it or is it not?” And it’s like I’m trying to figure it out […] now what I say has a psychosis ring around it (P20)

Facets of this include not feeling free to allow their minds to be unoccupied (“I always have to be doing something,” P1), losing a sense of being able to rely on themselves acting authentically (“I’m not able to make right decisions,” P19), and experiencing voices as out of control (P17): “at first, I did not really appreciate myself, I felt maybe like I’d lost it, um, I am losing my mind […] then I did not feel like a human being.”

#### The push and pull of dehumanising forces

Participants reported a wide range of forces moving them up and down these experiential continua. Particularly, dehumanising attitudes held by other people, which incorporated both animalistic and mechanistic dehumanisation, as well as being seen by people as uncanny, bizarre, or dangerous, had a powerful effect on how dehumanised voice-hearers felt in their interactions with other people, as illustrated by P4 when he said, “in the early phases I felt people were treating me as less than human […] somebody who was slightly irrational, a bit bizarre.” These attitudes connected to ostracism and stigma. For example, with regards to interactions with friends, P14 expressed, “they make me feel like I’m odd one out and I’m not a human […] in short, they dehumanise me,” and P15 highlighted, “I told my friend about my experience with the voices I hear and all she could tell me was that I was going crazy no such things existed and you see how a close friend can share something so delicate with and she turns out to tell you that it’s not possible […] so I tend to feel bad.”

There appeared to be a process whereby meta-dehumanisation became self-dehumanisation. Meta-dehumanisation was by voices, by other people, or both, as described by P15, “one criticises me, some encourage me […] but mostly they dehumanise me, they discourage me badly,” and P18, “I think the voices they are the same as the people, you know, and the society, I hate it.”

Many reported that verbal abuse, relentless pressure, and the omnipotence and malevolence of voices contributed to their feeling of being dehumanised:

Three evil men basically erm they kind of so they torment me sort of deride me […] the persecutory type of experiences and I think initially they almost had a sort of a god-like quality to them […] kind of all powerful and difficult to resist (P4)

Some reported experiencing a felt sense that voices deliberately and maliciously sought to compound trauma in their life, adding an additional layer to abuse, as indicated by P1, “the bad voices sort of capitalised on that and made my life even more of a misery.”

Participants also experienced several situations in which they observed society largely perceiving them as unwelcome or beyond hope, as well as situations where purported protectors perpetuated prejudice:

You tell people in the outside world that you’ve got mental health issues, that’s one thing. That’s one level of stigma. If you further admit that, by the way, I hear voices as well, then you get a lot of opprobrium directed at you […] people say that you must be a bad person, you’re dangerous […] we can’t trust what you say (P1)

#### Reclaiming life through humanising forces

Voice-hearers reported a range of forces which helped them to feel human again or retain their belief in their humanness in the first instance. These included consistent acceptance by others, which helped with feeling a sense of belonging and safety with other people, as P17 experienced with his family, “they understand how it is for me and they are always there and accepting my problem and maybe they give me so much love and understanding.”

Reclaiming personal agency and control over life through effective coping, engagement with meaningful activities, helping others earlier in their recovery from distressing voices, helped with progressively feeling human again for participants such as P1, “I’m living with myself as I am, and learning to cope with the voices, and perhaps more importantly teaching other people how to cope with the voices.”

Those who felt human had managed to develop a tolerance towards social rejection, a sense of being on their own side, or a perception of the voice-hearing experience as special and uniquely human, which was how P5 conceptualised it, “the fact that I can hear voices and my friends and maybe close relatives cannot hear […] I think it’s unique, but I have not had anything extraordinary that has made me feel inhuman, I just feel normal.”

Changes in voices or the perception of voices, such that they had diminished authority or were perceived as an event in the mind, was helpful in strengthening a sense of agency and regaining trust in self, as well as integrating the sensory experience of voices and gaining distance from the experience, as indicated by P10, “that was the big thing, like after I accept this thing, like you are able to control this, like you make yourself the controller” and by P4:

Re-assert some authority myself over them […] that kind of power dynamic is the key thing that’s evolved over time but initially I was absolutely terrified by these experiences […] I felt very unsafe both from myself and from my family (P4)

Recognition that abusive voices were immoral in their behaviour and recognition that voices might be trauma manifesting itself were powerful ways in which people began to make sense of the voice-hearing experience and respond to it in a more self-compassionate way. Similarly, engagement in safe group contexts helped with increasing belonging, as was the case for P4, “the more then that I was able to leave the house to feel safe again […] that sort of reintroduction to society meant that I was more kind of socially acceptable.”

Voices which were perceived as empowering, guiding, benevolent, were also identified as humanising forces, as P6 noted, “it was lack of trust, lack of support, people not being there, people who are pushy […] I think I had this kind of support from the voice.”

Those who held onto a sense of their humanness had stuck close to others who accepted and supported them with the challenges of hearing voices, as P10 emphasised, “they have been with me throughout uh it’s without them I do not know if I would be where I am like they were I say number one who came let me say to my rescue.”

Immersion in benign nature was helpful in enabling participants to feel human again and reducing the level of threat they experienced, as shared by P8, “being in safe place and nature […] time to be as safe as I could be […] that’s how I’m dealing with it.”

Belief and trust in a part of the self that was vital, permanently present in the background, and able to provide hope in coping with voices, was also important for participants in feeling human again, as shown by a metaphor P1 was taught by a psychologist, “I decided I would believe there is a little spark, a little light that never goes out, metaphorically speaking, inside you,” and by P12:

Not giving up on myself, even if others they gave up on me, and trying my level best to, even if they’re challenging, I kind of come up with a new challenge that it’s hard for them to overpower me on that, I think I have an upper hand now and I have an advantage over them (P12)

### External validation of themes

Themes and sub-themes were presented to a panel of Experts by Experience, who all had experience of distressing psychosis and linked experience of using mental health services. The group agreed with all the themes as presented, and shared personal experiences of dehumanisation pertaining to each. Subthemes of “Strength of Personal Agency” and “Sense of Belonging with Other Humans” resonated particularly strongly, as did experiences of meta-dehumanisation perpetrated by healthcare workers and members of society. The group emphasised the humanising capacity of peer support, connection with other people who understood their experience, and acceptance. It was felt that no new themes or subthemes should be added.

## Discussion

### The phenomenology of dehumanisation in voice hearers

The present study is the first to develop a conceptual framework grounded in subjective experience of self-dehumanisation in people hearing distressing voices. Central to this framework are six essentially human experiential continua which coalesce at the point of self-dehumanisation – that is, sensory fragmentation, belonging with other humans, self as a private and coherent entity, worth as a human being, personal agency, and trust in one’s own credibility and reliability. The six continua align with and extend the literature on voice-hearing drawn from those with and without psychosis diagnoses.

Losing trust in one’s own sensory and perceptual experience and credibility was identified as one such experiential continua. This echoes research showing how voice-hearers are often perceived as “unreliable narrators” and start to doubt their own credibility (30). Likewise, reduced sense of agency was highlighted across participants as an important dehumanising experiential continuum. Formanowicz et al. (31) found, in a general sample, that agency attributions are primary determinants of humanness attributions, such that where agency is not attributed, humanness is not. Losing a sense of being the author of one’s actions and lives has long been posited as central to a loss of hope in people experiencing psychotic phenomena such as auditory hallucinations, and increasing sense of personal agency is a key mechanism in recovery (32). Again, the present study suggests that fundamental to feeling dehumanised as a voice-hearer is destruction of a person’s sense of having a coherent sense of identity, and their self-worth. An essential element of our perception of ourselves as human is our being in possession of a self. A systematic review of qualitative research found that individuals experiencing psychotic phenomena including voice-hearing struggle to maintain a coherent sense of self, reflected in changes in narrative identity towards detached narration and disjointed events, underpinned by consistent suffering across life stages (33). Overall, loss of trust in oneself in different ways, appears to connect all six experiential continua, and highlights the dehumanising and deconstructive effect of this.

Some participants described feeling propelled towards the end of the continuum by voices, and difficult interactions and experiences, whereas for others it felt like a gradual erosion of one or more of the six essentially human experiential dimensions. Voice omnipotence, malevolence, and omniscience (18) were found to be powerful dehumanising forces; to a lesser extent, guiding and benevolent voices could be humanising forces. As is to be expected, societal prejudice and stigma emerged as dehumanising forces, and several participants reported perceiving some mental health professionals to embody these negative dehumanising attitudes. One powerful humanising force was a sense of belonging with others. Social psychological research shows how social ostracism closely relates to meta-and self-dehumanisation (5). In research on psychological therapy for distressing psychosis, universality (a recognition of shared humanity with others) emerges as the single most important therapeutic group factor in both cognitive therapy groups (25, 26) and in mindfulness-based groups (34, 35).

### Reflections on the continuum model

First proposed by Strauss (36), the continuum model posited that psychotic symptoms, such as auditory hallucinations or voice-hearing in clinical sample (samples lay on continua functions, which extend into the general population (37, 38). A continuum model underpins both cognitive therapy (39) and mindfulness-based therapies for psychosis (1). It contrasts with traditional psychiatric approaches, which emphasised discontinuity from other human experience and framed psychotic phenomena such as voice-hearing as lying on the far side of an ‘abyss’. The continuum model raises an interesting question about the positive symptoms of psychosis – that is, if at the near end lie everyday counterparts to voices (e.g., (40)) and persecutory beliefs (e.g., (41)), then what lies at the far – that is, clinical – end of the continuum? In the present study, distressing voices certainly emerged as powerful forces of both meta-dehumanisation and self-dehumanisation, but they appear along the six experiential continua described. For the participants in the present study, distressing voices was not the end of the line – it was self-dehumanisation that lay at the end of their experiential continua.

The sample in the present study is not psychosis specific. Rather, the intention was to recruit a diverse sample of voice hearers from across the voice-hearing continuum, and it is therefore important not to assume that the presence of voices automatically implies the presence of psychosis. Moreover, it is important for voice hearers themselves that the experience is not automatically pathologized as a symptom of, or sign of vulnerability to, psychosis or schizophrenia. However, there is evidence that the findings in the present study on self-dehumanisation have relevance to the experience of voice-hearing across the continuum, including those hearing voices with psychosis. All participants described voice-related distress, in line with the inclusion criterion: and voice-omnipotence, malevolence and resistance were strongly reported by many participants, in many instances at high levels. Although at no point in the study was service-use asked about, we know at least four of the sample had used mental health services linked to voices (e.g., one person said voices destroyed their life, with three hospital admissions in a year). It is impossible to know how many others had also done so. All five experts by experience on the service user consultation panel had experienced service-use for psychosis, and all five endorsed the core theme and sub-themes, adding illustrative examples from their own lives. Nonetheless, further research with psychosis-specific samples is needed to establish the degree to which experiences of self-dehumanisation characterize the extreme end of the psychosis continuum.

### Strengths and limitations

A strength of this study was that participants exhibited a wide range of ages and ethnicities and an almost even balance of binary genders. This may enhance the transferability of the findings to other contexts. In keeping with a critical, realist contextual stance, the present research accepts as real the voice hearers’ lived experience. However, the sample was obtained by convenience and limited to those self-identifying as hearing distressing voices. It is important to emphasise once again that no diagnostic assessment was undertaken, the sample was not psychosis-specific, nor was the range of symptoms of psychosis assessed. Participants described a range of current levels of distress and disturbance, with some seeming to be further along in their recovery journey. Crucially, all 20 participants could relate to and describe experiences of self-dehumanisation. Future studies would benefit from sampling participants with a diagnosis of psychosis and a range of positive and negative symptoms to assess generalisability of the current findings. A final strength of the present research was the external validation of results. Checking the findings with an independent panel of experts by experience demonstrates that the findings have relevance beyond the initial study population and reinforces the argument for the relevance of the findings to people with psychosis.

### Implications and future directions for theory, research, and practice

Dehumanisation is emerging as an important new trans-diagnostic concept in understanding mental health problems. Future research and practice can explore the relevance of the phenomenology of self-dehumanisation in voice-hearers to other clinical groups. In relation to psychosis, research can examine how self-dehumanisation relates to social isolation, depression, and suicidality, as well as other symptoms within psychotic experience. The six essentially human continua might also inform development of much-needed measures of self-dehumanisation to support research and practice in mental health.

The concept of self-dehumanisation also adds a potentially important nuance to our wider understanding of self-compassion (42), an important mechanism of change in contemporary mindfulness-based therapies. One of three characteristics of Neff’s definition of self-compassion is ‘common humanity versus isolation’. It might be argued that self-dehumanisation is the most extreme expression of isolation, or separation from our common humanity. Thus, at its extreme the characteristic could be framed as ‘common humanity versus isolation and self-dehumanisation’.

Self-dehumanisation may have important implications for the ongoing evolution of psychological therapies for distressing voices in psychosis. One of the defining attributes of clinical cognitive and mindfulness-based approaches to psychosis has been an ever-greater emphasis on the concept of the self. It has long been recognised that psychotic symptoms such as voices and paranoia are linked to low self-esteem (e.g., (43, 44)) and cognitive therapy includes experiential methods specifically designed to work with the self-concept (e.g., (35)). Whilst profoundly low self-esteem (or negative schema) is acutely distressing, it is at least an identity – participants in the present study described a point beyond extremes of low self-esteem, where in self-dehumanisation the very sense of having a self or identity at all is lost. Future research might examine if and how psychological therapies for psychosis address feelings of self-dehumanisation and the six experiential continua in particular.

Specifically, mindfulness for psychosis has been described as a humanising therapeutic process (1) and the present study gives an indication of how mindfulness for psychosis may be well suited to address the challenges posed by self-dehumanisation. The focus on awareness of moment-to-moment experience offers a direct means with which to reconnect with and rebuild trust in sensory experience. Also, choosing to allow voices, thoughts, and images to come fully into awareness, is known to restore a sense of personal agency and power (45). Again, grounding oneself in decentred awareness of psychotic experience, rather than being lost in it, helps restore a sense of coherence to the self as someone who can both feel and observe what is happening.

Finally, in relation to group therapies for psychosis, it has been argued that non-specific therapeutic group factors play an important role (46). In the present study, the experiential continua of ‘sense of belonging with other humans’ directly points to the transformational potential of groups for people with psychosis. Viewed through a lens of dehumanisation, it is striking that in research involving people with distressing psychotic voices, in both group cognitive therapy (25, 26) and group mindfulness-based therapy (34, 35), group members rated universality (learning that I am not alone, that others have similar experiences) as the most subjectively important of eight primary group factors. Again, a Grounded Theory of the process of change during mindfulness for psychosis groups placed ‘discovering that I am not different’ at the very end of the transformational process (45). Thus, universality is emerging as a central humanising force within therapeutic groups for psychosis and may be a target for future research examining what can mitigate experiences of dehumanisation in those with psychosis.

## Conclusion

There can surely be no more profound a psychological threat than to lose a sense of being a person, of being human. For the voice hearers in the present study, experiences of self-dehumanisation were identified as the end of the line, an experience where six essentially human continua coalesce. The phenomenology of self-dehumanisation in voice hearers may add to our understanding of what lies at the far, clinical end of the continuum of psychotic phenomena, and provide a platform for further evolution of psychological therapies for psychosis.

## Data availability statement

The original contributions presented in the study are included in the article/Supplementary material, further inquiries can be directed to the corresponding author.

## Ethics statement

This study involving humans was approved by University of Bath Psychology Research Ethics Committee. The studies were conducted in accordance with the local legislation and institutional requirements. The ethics committee requirement for written informed consent from participants was met.

## Author contributions

BO’B-V and PC conceptualized, designed, and wrote up the study. BO’B-V completed the interviews, transcription and led on analysis. TJ completed the review of themes with an Expert by Experience panel. All authors contributed to the article and approved the submitted version.

## Footnotes

^1^ https://www.qualtrics.com/
