# Supplementary material for: Self-dehumanisation in voice hearers: the end of a continuum
Source: Front Psychiatry. 2023 Oct 3;14:1173380. doi: 10.3389/fpsyt.2023.1173380 (PMC10579558; doi:10.3389/fpsyt.2023.1173380)
Supplement: Supplementary file 1 [file Data_Sheet_1.DOCX]

**Supplementary Materials A: Semi-Structured Interview Schedule**

1. Please could you start by telling me a bit about what your experience of hearing a voice or voices is like?
2. How has hearing voices affected how you live your life?
3. How has your experience changed over time?
4. Do you think hearing voices has changed your experiences of how other people see you?
   1. In what ways?
   2. How else has it changed how people are with you?
   3. How have those experiences made you feel?
5. How has hearing voices changed how you view yourself?
   1. In what ways?
   2. How else has it changed how you see yourself?
   3. Have there been any positive changes?
6. Have you ever felt that other people view you as less of a person because you hear voices?
   1. What experiences have contributed to this?
   2. How have you reacted to and responded to these experiences?
7. Has hearing voices ever made you feel less of a person?
   1. In what ways?
   2. Can you tell me more about that?
8. Has that feeling of being less of a person changed?
   1. How does that feeling affect you day-to-day?

**Supplementary Materials B: Summary of Themes, Subthemes, and Codes.**

**Table 1**

*Summary of themes, subthemes, and codes*

| Themes | Subthemes | Codes |
| --- | --- | --- |
| Dehumanisation as the End of Experiential Continua | “It’s like Burning Alive”: Extent of Distressing Sensory Fragmentation | Enhanced concentration due to managing voices  Entrapped by voice hearing  Grounding to let the wave of voices pass  Inexplicability of the sensory experience  Perceiving self as having alien or bizarre sensory experiences  Sensory intensity of distress related to voice-hearing  Voices as harmless events of the mind |
|  | “You Belong to the Wild and They Belong to the Earth”: Sense of Belonging with Other Humans | Concealment of voice-hearing from others  Feeling alone with the experience of voices  Feeling defeated by multiple exclusions  Feeling insignificant in interactions with services  Feeling judged regardless of disclosure  Feeling not important enough to receive help  Feeling you should remove yourself from humanity  Feeling unable to relate to others  Feeling unable to trust others enough to disclose  Feeling unacceptable to others  Intersection of race with mental health as a doubly disconnecting stigma  Loss of social information  Perceived risk of abandonment from humanity  Reduced social risk-taking  Sticking close to others to protect self from voices  The dark bottom of the hierarchy of beings |
|  | “They Destroy Your Sense of Self”: Integrity of Self as a Private, Coherent Entity | Deconstruction of sense of self  Disparity between own narrative and recorded narrative in services  Erosion of identity  Fear of exposure due to voices being audible  Impact of loss of work on identity  Incongruence between voices and self  Loss of privacy of own mind  Personality has been diminished by voice-hearing  Voice-hearing obscures knowing yourself  Voices alter both valued and devalued personal qualities  Voices help to know yourself better |
|  | “I’m Part of the Scrap Heap”: Sense of Worth as a Human Being | Conceding to verbal abuse of voices  Constant fight to prove own worth  Feeling a mismatch with gendered societal expectations due to impact of voices  Feeling like a worthless person  Feeling your best is not enough to count you as human  Feelings of inadequacy relative to others  Felt pain of not enough-ness  From meta- to self-dehumanization  Loss of dignity  Moral distress related to voices  Society’s hierarchy of beings as immutable  Voice-hearing as a uniquely human capability  Expectations of others of greater than ordinary capabilities |
|  | “They Took a Part of Me”: Strength of Personal Agency | Concealment of true self constrains functioning  Defeat when trying to build a successful life  Feeling unable to get it right  Futility of fighting the voices  Impact of voices on ability to function  Loss of control and agency over life  Ongoing attempts to fight voices  Unable to meet voices’ expectations  Voices as barrier to meeting own expectations  Voices driving values incongruent action  Voices as empowering and protective |
|  | “It has a Psychosis Ring Around it”: Trust in Own Credibility and Reliability | Loss of reliance on self to act authentically  Loss of trust in own credibility  Need to downplay experience to others  Not being free to allow mind to be unoccupied  Shock of voice-hearing coming out of nowhere  Voice-hearing as an experience out of own control  Improved coping with voices over time  Retention of trust in yourself |
| The Push and Pull of Dehumanising Forces |  | Absence of genuine acceptance from others  Attitude of voices – questioning your credibility, trustworthiness, decision-making capacity  Attitude of others – being seen as uncanny, bizarre, or dangerous  Attitude of others – questioning your credibility, trustworthiness, decision-making capacity  Being taken advantage of by others  Belief of voices and others – you lack agency  Cultural beliefs about voice-hearing as deviant  Dehumanising content of voices’ speech  Dehumanising messages in socio-political actions  Discreditation through invisibility of voice-hearing  Doubt in your credibility as a weapon  Experience of services compounding trauma  Lack of witness to trauma  Meta-dehumanisation as an observable category change others’ make  Ostracism from family members and friends  Own perception of wider society – increasing discrimination  Own perception of wider society – unwilling to understand  Perception of others – you are a different person now  Perception of others – you are beyond change or hope  Perception of others – you lack warmth  Perception of others – you are less able than the average human  Prejudice unchallenged by purported protectors  Relentless omnipotent and malevolent voices  Social rejection and stigma  Verbal abuse by voices  Voice-related meta-dehumanization contributing to other-related meta-dehumanization  Voices as a source of pressure  Voices compounding moral distress  Voices maliciously compounding trauma  Voices and others agree on criticisms |
| Reclaiming Life through Humanising Forces |  | Acceptance invokes greater control over voices  Ambivalence about voice-hearing  Change in voice origin beliefs  Consistent valuing and acceptance by important others  Determination to prove others’ dehumanising attitudes wrong  Devaluation of the judgement of voices and others  Distance from voice-hearing through humour  Empowerment through meaningful work and activities  Exercising choice in response to voices  Experience of transformational moments  Exposure of voice-hearing to aid others  Finding a fresh start  Forging common humanity and hope with peers  Immersion in benign nature  New possibilities for personal achievement  Non-judgemental content of voices  Others reframing voice-hearing experience  Positive experiences of disclosure  Raising awareness of voice-hearing to help others  Reciprocity with the natural world  Reclaiming authority and agency  Recognition of own human rights  Recognition of current persecution as manifestation of past trauma  Recognition of the immorality of abusive voices  Recognition of positive qualities of other voice-hearers  Reintegration and belonging with others  Religious practice as source of strength  Safety of a group  Self-acceptance and self-appreciation  Successful coping with voices  Tolerating standing alone  Voice-hearing as source of support for decision-making  Voices as protection from loneliness  Voices having or developing lower authority  Withdrawal for self-protection  Withdrawal to regain control over voices |
